# Appetite-preserving gastrectomy (APG) for esophagogastric junction cancer: preserving the residual stomach as an endocrine organ

**DOI:** 10.1007/s10120-025-01603-z

**Published:** 2025-03-18

**Authors:** Naoki Hiki, Tadashi Higuchi, Koshi Kumagai, Kota Okuno, Hiroyuki Minoura, Yumi Sato, Shohei Fujita, Hiroki Harada, Motohiro Chuman, Marie Washio, Mikiko Sakuraya, Masahiro Niihara, Yusuke Kumamoto, Takeshi Naitoh, Keishi Yamashita

**Affiliations:** 1 Department of Upper Gastrointestinal Surgery, Kitasato University School of Medicine, 1-15-1 Kitasato, Minami-ku, Sagamihara, Kanagawa 252-0374 Japan; 2 Department of Nutrition, Kitasato University Hospital, Sagamihara, Japan; 3 Department of General-Pediatric Hepato Biliary Pancreatic Surgery, Kitasato University School of Medicine, Sagamihara, Japan; 4 Department of Lower Gastrointestinal Surgery, Kitasato University School of Medicine, Sagamihara, Japan; 5 Division of Advanced Surgical Oncology, Research and Development Center for New Medical Frontiers Department of Upper Gastrointestinal Surgery, Kitasato University School of Medicine, Sagamihara, Japan

**Keywords:** Appetite-preserving gastrectomy, Esophagogastric junction cancer, Ghrelin secretion, Lean body mass preservation, Quality of life after gastrectomy

## Abstract

**Background:**

Loss of appetite following gastric cancer surgery, particularly total gastrectomy, significantly impacts patient quality of life due to the removal of the ghrelin-secreting region. We developed appetite-preserving gastrectomy (APG), a modified total gastrectomy that preserves this region.

**Methods:**

Ten consecutive patients with esophagogastric junction cancer who were indicated for total gastrectomy and underwent APG between April 2023 and April 2024 were evaluated for early surgical outcomes, appetite, and changes in weight and body composition.

**Results:**

There were no postoperative complications of grade II or higher (Clavien–Dindo classification). Appetite, assessed using the Simplified Nutritional Appetite Questionnaire, showed no significant impairment at 3 months (14.5 points, *P* = 0.82) and 6 months (15 points, *P* = 0.44) postoperatively compared with preoperative values. Oral calorie intake was maintained at 3 months (1675 kcal, *P* = 0.97) and 6 months (1675 kcal, *P* = 0.22) postoperatively compared with preoperative levels. The patients’ body weight decreased by 9.2% at 6 months postoperatively compared with preoperative values, but their lean body mass remained stable. Although a significant decrease in the blood Ghrelin levels was observed postoperatively, 53% and 60.4% of the preoperative levels was maintained at one month and 6 months, respectively.

**Conclusions:**

APG is a safe procedure that preserves the residual stomach as an endocrine organ, maintains ghrelin secretion and appetite, and prevents muscle loss. However, further trials are required to compare the efficacy of APG with total gastrectomy in preventing postoperative appetite loss.

## Introduction

After gastric cancer surgery, patients often suffer from appetite loss and reduced food intake [1]. This loss of appetite significantly reduces their quality of life, especially after total gastrectomy [2, 3], and a method to ameliorate this symptom either medically or surgically had been one of the unmet needs in the management of gastric cancer.

The reasons for the decreased oral intake among patients after gastrectomy may include: 1) reduced residual stomach volume, 2) decreased peristalsis and other gastric functions, and 3) lack of secretion of ghrelin, a hormone that regulates appetite. Total gastrectomy results in all of these dysfunctions, leading to considerable weight loss. Postoperative weight loss negatively affects the adherence to postoperative chemotherapy for gastric cancer and has prognostic implications [4–6]. Ghrelin is an endogenous ligand of the growth hormone secretagogue receptor (GHS-R), a peptide hormone produced by the stomach [7]. It acts on the pituitary gland to stimulate growth hormone (GH) secretion and on the hypothalamus to stimulate appetite [8]. Ghrelin production is mainly carried out by endocrine cells called X/A-like cells, which are distributed from the fornix to the upper body of the stomach [9]. Therefore, gastric resection of this region, such as in total or proximal gastrectomy (PG), results in considerable loss of appetite and body weight [2, 3].

Various function-preserving gastrectomy techniques, including pylorus-preserving gastrectomy (PPG), PG, and subtotal gastrectomy, have been developed [10]. However, attempts to develop function-preserving gastrectomy techniques that focus on preserving gastric volume have not yielded satisfactory outcomes. A technique involving the addition of a jejunal pouch to a total gastrectomy was previously developed, but it did not effectively prevent weight loss [11]. In subtotal gastrectomy preserving the ghrelin secretory area, the residual stomach was only 30%, whereas in PG resecting the ghrelin secretory area, the residual stomach was approximately 70% [12]. Nevertheless, the advantage in terms of the volume of residual stomach did not translate into preservation of body weight among patients who underwent PG. These findings implicate that resection of the ghrelin secretory area is an intrinsic determinant of weight loss.

Therefore, we developed appetite-preserving gastrectomy (APG), a novel surgical procedure that preserves the gastric ghrelin secretory area, allowing the remnant stomach to function as an endocrine organ. This procedure is indicated for patients with upper gastric or gastroesophageal junction cancers who are candidates for total or proximal gastrectomy. In this feasibility study with a planned sample size of 10 patients, we prospectively evaluated the safety and short-term outcomes of APG performed by an open approach.

## Materials and methods

### Patients

Patients between 20 to 85 years of age with EGJ cancer with an esophageal invasion length of ≤ 3 cm were eligible. The EGJ was identified as the lower margin of the palisading small vessels on endoscopy according to the Japanese Classification of Esophageal Cancer, 11th Edition [13]. Tumors of any histological type with a depth of up to cT4a and preoperative lymph node metastasis up to cN2 with no distant metastases who would otherwise be indicated for either total or proximal gastrectomy were included. However, cases where metastasis to the No. 4sa lymph node (No. 4sa LN, also known as left greater curvature LNs along the short gastric arteries) or No. 10 lymph node (No. 10 LN, also known as splenic hilar LNs) was suspected before surgery were excluded. Additionally, while there was no restriction on tumor size, the tumor location in the stomach had to be predominantly in the lesser curvature with no obvious invasion of the anterior or posterior walls. Therefore, lesions with unclear borders such as Borrmann Type 4 or relatively large Type 3 cancers were excluded. Clinical data collected from the patients’ medical records included sex, age, tumor diameter, length of esophageal invasion, Siewert classification, histological type, staging, surgery-related factors, and postoperative complications. Staging was assessed using the Union for International Cancer Control TNM Classification of Malignant Tumours, 8th Edition [14]. Postoperative complications were categorized based on the Clavien–Dindo classification [15]. This trial is registered with UMIN-CTR (number: UMIN000053011) and was conducted with the approval of the institutional review board (C22-083).

### Surgical procedure

**Fig. 1:**
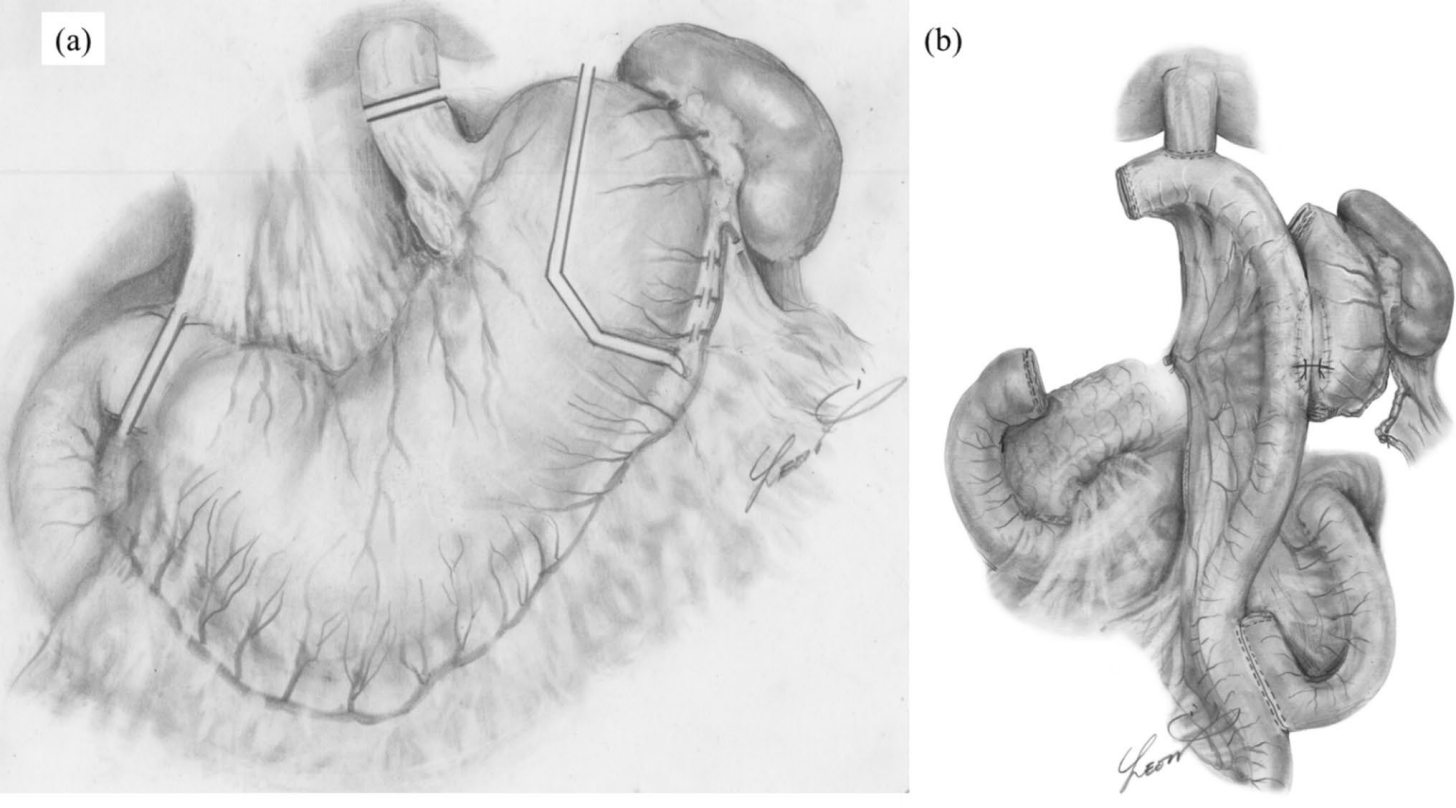
**a.** Gastric transection for APG: After completion of the lymph node dissection, the duodenum was transected, followed by transection of the esophagus. A longitudinal gastric transection line was determined from outside the fornix No.2 lymph node region, and the line of dissection was determined where this intersected the gastrectomy line drawn from the anastomosis of the left epigastric artery and short gastric artery. **b** Reconstruction for APG: The esophago-jejunal anastomosis was performed using a circular stapler. The residual stomach and the elevated intestine were anastomosed using a linear stapler. The jejunal-jejunal anastomosis of the Rouen-Y anastomosis was then performed by conventional methods

Since APG is a novel surgical procedure, surgery in this trial was performed exclusively by an open approach to ensure appropriate placement and balance of the residual stomach and jejunum after gastrectomy. Lymph node dissection was performed as described in the Japanese Classification of Gastric Carcinoma (October 2017, 15th edition) by the Japanese Gastric Cancer Association [16]. However, No. 4sa and No. 10 lymph nodes were not dissected, the left gastroepiploic artery was divided, and the short gastric arteries served as the primary blood supply to the residual stomach (Fig. 1a).

After lymph node dissection, the duodenum was transected, followed by esophageal transection. The extent of the gastric portion to be preserved for APG was marked. A longitudinal gastric transection line was determined outside the fornix No. 2 lymph node (No. 2 LN, also known as left paracardial LNs) region, intersecting with the gastrectomy line drawn from the anastomosis of the left gastroepiploic and short gastric arteries. The size of the stomach to be preserved as ghrelin secretory area was approximately 6 cm × 10 cm. A portion of the resection margin nearest to the tumor was sampled from the resected specimen after gastrectomy and sent to the pathologic examination. To evaluate the adequacy of blood flow in this area, we administered 2 ml (2.5 mg/ml) of indocyanine green (ICG) intravenously after dissolving 10 ml of undiluted ICG. The remaining stomach was observed for approximately 30 s after the ICG injection to assess blood flow adequacy. After confirming sufficient blood flow, gastrectomy was performed using a linear stapler. Blood supply to the remaining stomach is likely to derive from the short gastric artery and the esophago-cardiac branch of the left diaphragmatic artery.

The esophagojejunal anastomosis was performed using a circular stapler, ensuring that the tip of the elevated intestine was facing the right side. A stapler insertion opening was made in the posterior wall of the lower caudal side of the residual stomach and anastomosed with the anterior wall of the elevated intestine using a linear stapler. The insertion hole was closed with a continuous suture. Jejunal–jejunal anastomosis of Roux-en-Y was performed using conventional methods (Fig. 1b). Intraoperative endoscopic examination was performed to confirm the anastomosis. Observation of the remaining stomach was possible in all cases.

### Appetite assessment after APG

The Simplified Nutritional Appetite Questionnaire (SNAQ) was used to assess appetite after APG [17–19]. SNAQ scores were measured preoperatively and at 1 month (POM1), 3 months (POM3), and 6 months (POM6) postoperatively. The SNAQ consists of four questions (appetite, satiety, taste, and number of meals per day), each rated on a five-point scale. The total score ranges from 4 to 20, with lower scores indicating reduced appetite. Study participants with SNAQ scores < 14 were classified as having poor appetite. To minimize bias from the Hawthorne effect, the questionnaire was administered by an outpatient dietitian. This study also compared changes in caloric intake and serum values of total protein and albumin at four-time points: preoperatively, POM1, POM3, and POM6.

### Perioperative body composition changes after APG

Body weight and lean body mass (LBM) were measured preoperatively and at POM1, POM3, and POM6. Body composition was assessed using an InBody S10 body composition analyzer (InBody Co., Ltd., Seoul, Korea) in the sitting position.

### Des-Acyl Ghrelin level measurement after APG

Blood samples were collected in ethylenediaminetetraacetic acid (EDTA) and aprotinin-containing tubes (SRL, Inc., Tokyo, Japan). The samples were centrifuged at 1500 × g for 15 min, and after plasma separation, they were stored at − 80 °C until des-acyl-ghrelin levels were assessed. Des-acyl-ghrelin levels were measured using a commercially available enzyme-linked immunosorbent assay (ELISA) kit (SCETI Co., Ltd., Tokyo, Japan) following the manufacturer’s instructions. The ELISA kit had a detection range of 13.6–870 fmol/mL. Measurements were performed preoperatively and at POM1, POM3, and POM6. Blood samples were collected after fasting.

### Statistical analysis

Data are presented as medians and ranges. The Wilcoxon rank-sum test was used for comparisons of continuous variables, and Fisher’s exact test was used to analyze differences between groups. All statistical analyses were performed using JMP® 16 software (SAS Institute Inc., Cary, NC, USA). A two-tailed P-value < 0.05 was considered statistically significant.

## Results

### Patient Characteristics

Table 1 presents the characteristics of the patients with EGJ cancer (n = 10). Age, sex, American Society of Anesthesiologists physical status, Siewert classification, length of esophageal invasion, clinical stage, histological type, and chemotherapy status were assessed. For the overall clinical staging, 4 patients (40%) had stage I disease, 1 patient (10%) had stage II disease, 4 patients (40%) had stage III disease, and 1 patient (10%) had stage IVA disease. Altogether, five patients (50%) received neoadjuvant chemotherapy.

**Table 1:** Patient characteristics

Variables	N = 10 (%)	
Sex		
Male / Female	5 /5	(50% / 50%)
Age (year) median (range)	65	(44–82)
ASA-PS		
1 / 2 / 3	0 / 10 / 0	(0% / 100% / 0%)
Siewert Type		
II / III	9 / 1	(90% / 10%)
LEI (mm) median (range)	10	(0–20)
Clinical T status		
1 / 2 / 3 / 4a	3 / 2 / 3 / 2	(30% / 20% / 30% / 20%)
Clinical N status		
0 / 1 / 2	6 / 3 / 1	(60% / 30% / 10%)
Clinical M status		
0 / 1	10 / 0	(100% / 0%)
Clinical Stage		
I / II / III / IVA	4 / 1 / 4 / 1	(40% / 10% / 40% / 10%)
Histology		
Adenocarcinoma (adca) / Squamous cell carcinoma	9 / 1	(90% / 10%)
Well differentiated tubular adca. (tub1)	6	(60%)
Moderately differentiated tubular adca. (tub2)	2	(20%)
Poorly differentiated adca. (por)	2	(20%)
Neoadjuvant chemotherapy	5	(50%)

T/N/M status and stage were according to the Union for International Cancer Control (UICC)

TNM classification of malignant tumors (8th edition). *LEI* length of esophageal invasion

### Surgical Outcomes and Pathological Findings after APG

Table 2. The median operative time was 459 min, and the median estimated blood loss was 632 ml. In one case (10%), cancer was diagnosed based on the finding of an intraoperative frozen section of the esophageal proximal resection margin. However, after additional resection of the esophageal proximal margin, the diagnosis was ultimately negative. Moreover, the intraoperative frozen section of the margin of the preserved ghrelin secretory area nearest to the tumor was examined and was negative in all cases. Consequently, R0 resection was accomplished in all cases. None of the patients had postoperative complications of grade II or higher according to the Clavien–Dindo classification. There was no postoperative mortality, and the median length of the postoperative hospital stay was 9 days. Table 3 shows the pathological findings, which were as follows: stage I in five cases (50%), stage II in two cases (20%), stage III in two cases (20%), and stage IV in one case (10%). The median tumor size based on the pathological specimens was 24 mm, and the histology of the pathological specimens was similar to that of the preoperative diagnosis. In all cases, R0 resection was achieved and the proximal margin was negative. Additionally, regarding adjuvant chemotherapy, three patients received postoperative adjuvant chemotherapy. Among them, two patients received S-1 (TS-1®) monotherapy, while one patient received DS therapy (docetaxel + S-1). Throughout the observation period, all patients continued treatment without dose reduction.

**Table 2:** Early postoperative surgical outcomes

Variables		*N* = 10	
Operative time (min)		459	(354–526)
Estimated blood loss (ml)		632	(75–1692)
Initial intraoperative frozen section of the proximal margin			
Positive for cancers / Negative for cancers		1 / 9	(10% / 90%)
Secondary additional intraoperative frozen section of the proximal margin			
Positive for cancers / Negative for cancers		0 / 10	(0% / 100%)
Intraoperative frozen section of the remnant stomach side margin			
Positive for cancers / Negative for cancers		0 / 10	(0% / 100%)
Anastomotic leakage		0	(0%)
Pancreatic fistula		0	(0%)
Any Postoperative complication (*Grade III or above*)		0	(0%)
Anastomotic leakage		0	(0%)
Pancreatic fistula		0	(0%)
Mortality		0	(0%)
Postoperative hospital stay (day)		9	(7–15)

Date expressed as median (range)

Postoperative morbidity analyzed according to Clavien-Dindo classification

**Table 3:** Pathological findings

Variables	*N* = 10	
Pathological T status		
1 / 2 / 3 / 4a	6 / 2 / 1 / 1	(60% / 20% / 10% / 10%)
Pathological N status		
0 / 1 / 2	7 / 0 / 3	(70% / 0% / 30%)
Pathological M status		
0 / 1	10 / 0	(100% / 0%)
Pathological Stage		
I / II / III / IVA	5 / 2 / 2 / 1	(50% / 20% / 20% / 10%)
Tumor size (mm)	24	(6–48)
Histology		
Adenocarcinoma (adca) / Squamous cell carcinoma	9 / 1	(90% / 10%)
Well differentiated tubular adca. (tub1)	3	(30%)
Moderately differentiated tubular adca. (tub2)	3	(30%)
Poorly differentiated adca. (por)	3	(30%)
Mucinous adca. (muc)	1	(10%)
Residual tumor		
R0 / R1	10 / 0	(100% / 0%)
Proximal margin (pPM)		
PM0 / PM1	10 / 0	(100% / 0%)
Proximal margin (mm)	9	(5–27)

Date expressed as median (range)

T/N/M status and stage were according to the Union for International Cancer Control (UICC)

TNM classification of malignant tumors (8th edition)

## SNAQ Score after APG

**Fig. 2:**
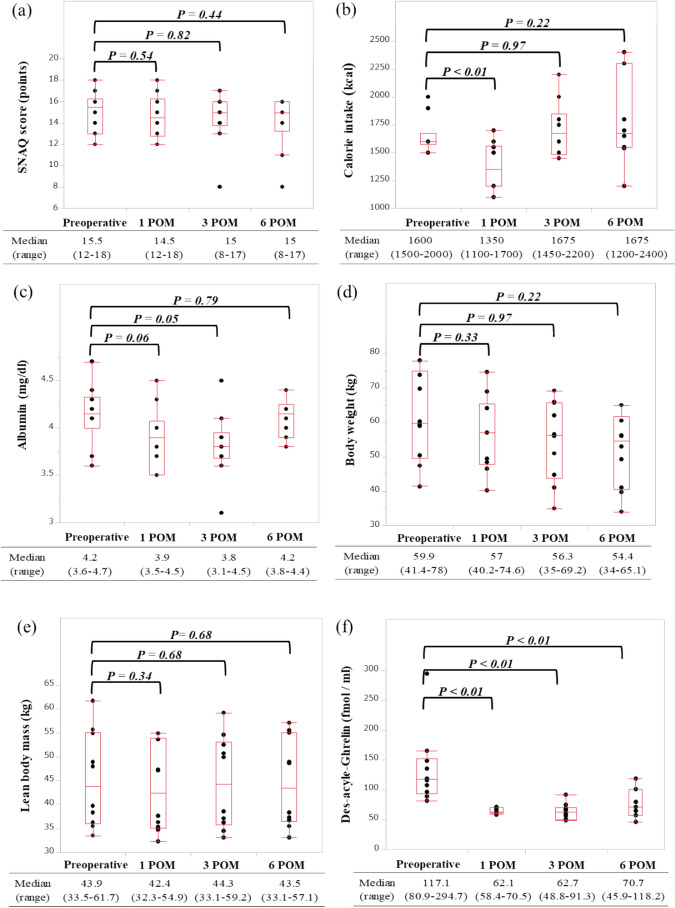
**a** Changes in Simplified Nutritional Appetite Quetionnaire (SNAQ) score after APG, **b** Changes in calorie intake after APG, **c** Changes in serum albumin levels after APG, **d** Changes in body weight after APG, **e** Changes in body weight lean body mass after APG and **f** Changes in body weight lean body mass after APG

The SNAQ scores are presented in Fig. 2a. The median scores were 14.5 at POM1, 15 at POM3, and 15 at POM6, showing no impairment throughout the 6 months. However, there was one individual with low scores at POM3 and POM6.

### Postoperative Changes in Caloric Intake, and Serum Albumin Levels and Total Protein Levels after APG

The median oral caloric intake decreased from 1600 kcal preoperatively to 1350 kcal at POM1, but recovered to 1675 kcal at POM3 and remained at 1675 kcal at POM6 **(**Fig. 2b**)**. Serum albumin levels decreased after surgery, but recovered to the preoperative level by POM6 **(**Fig. 2c**)**. The median total protein levels fluctuated from 6.6 mg/dl preoperatively to 6.5 mg/dl at POM1 and 7.0 mg/dl at POM6, with no significant differences (P = 1.00 and 0.25, respectively).

### Postoperative Changes in Body Weight and LBM after APG

Compared with the median preoperative weight of 59.9 kg, the patients’ body weights decreased consistently to 57.0 kg (− 4.8%) at POM1, 56.3 kg (− 6.0%) at POM3, and 54.4 kg (− 9.2%) at POM6 **(**Fig. 2d**)**. LBM at POM1 decreased to 42.4 kg (− 3.4%) compared with the preoperative value of 43.9 kg, but there was no further decrease, and the median value recovered to the preoperative status by POM3 **(**Fig. 2e**)**.

### Postoperative Changes in Ghrelin Parameters after APG

To preserve ghrelin as an endogenous hormone in post-gastrectomy cases, APG was developed in this study. Serum ghrelin levels were measured preoperatively, at POM1, POM3, and POM6, and showed a significant decrease at POM1, POM3, and POM6 compared to the preoperative value (P < 0.01 for all). However, 53% of the preoperative ghrelin levels were preserved at POM1, 53.5% at POM3, and 60.4% at POM6 **(**Fig. 2f**)**.

## Discussion

Appetite loss and subsequent weight and muscle volume losses after gastrectomy, especially after total gastrectomy, are associated with considerable postoperative impairment of the patients’ quality of life [1–6]. Appetite is assumed to be dependent on the size of the residual stomach after gastrectomy, but subtotal gastrectomy has been shown to preserve appetite and cause considerably lesser weight loss than total gastrectomy, even though only 30% of the stomach is preserved [10, 11]. This may be attributed to the fact that subtotal gastrectomy preserves the ghrelin-secreting region by preserving the greater curvature of the upper gastric body and fornix. In this study, we developed APG, a new gastrectomy technique, to preserve the patient’s appetite. In this new technique, fornix and greater curvature of the upper gastric body referred to as the ghrelin-secreting region by Takiguchi et al. [9], are preserved carefully so as not to impair curability. The residual stomach is anastomosed with the elevated jejunum, but no food passes into the residual stomach. In other words, the residual stomach is not intended to improve food retention capacity but theoretically serves as an endocrine organ that releases ghrelin into the bloodstream.

This study aimed primarily to assess short-term surgical outcomes of the APG, and we now consider that it is a safe procedure, given that no serious or unexpected complications were observed, and mortality was 0%. Although the study population, variation in the surgical approach, and period of patient accrual are quite different, the rates of serious anastomotic leakage and mortality were 11.9% and 0.4%, respectively, in a prospective nationwide multicenter observational study on EGJ cancer in Japan [20, 21]. On the contrary, our operative time was relatively long because of the use of ICG for assessing residual gastric blood flow, careful determination of the dissection line, and intraoperative rapid frozen pathology diagnosis, which were time consuming. Regarding the estimated volume of blood loss, one case of splenic capsular bleeding occurred during the manipulation of the splenic hiatus, suggesting that careful attention should be paid to handling the residual stomach, which adheres to the spleen.

Postoperative pathology showed that 50% of patients had stage ≥ III disease. Nevertheless, R0 and PM0 resections were achieved in all cases, indicating so far that our approach was oncologically safe. The only lymph nodes attached to the remnant stomach as ghrelin-secreting regions in the APG were No. 4sa LN and No. 10 LN, whereas No. 2 LN were completely dissected along with the stomach. According to a report by Kurokawa et al. [20, 22] on the lymph node metastasis rate in EGJ cancers, the metastasis rate of No. 4sa LN was 4.2%, suggesting that the possibility of lymph node recurrence around the preserved portion of the stomach is minimal. Furthermore, we could propose additional intraoperative assessment techniques such as submucosal injection of indocyanine green (ICG) for selective lymph node dissection and intraoperative rapid pathological evaluation to more precisely assess the extent of lymph node metastasis before proceeding to APG. Further studies with long-term follow-up are required to confirm the oncological validity of APG.

The presence or absence of appetite after the APG was evaluated using the SNAQ. In most published studies, good appetite was defined as an SNAQ score of ≥ 15 points [17–19]. In our study, the median preoperative SNAQ score was 15.5, and median score of ≥ 15 points was achieved at both POM3 and POM6, indicating that APG did not decrease the patients’ postoperative appetite. It must be noted that there was one patient with extremely low SNAQ score throughout the 6 months of follow-up, suggesting that APG may not always fulfill the expectations. The patients’ calorie intake decreased by 16.4% at POM1 but recovered to the preoperative value by POM3. This is in stark contrast with a previous report that the dietary intake after total gastrectomy decreased by 15.6% by POM1 and 5.3% at POM3, respectively, and the caloric intake did not completely recover even after 3 months [1]. Regarding nutritional parameters, serum albumin and total protein levels, which reflect the patient’s long-term nutritional status, were comparable with the preoperative values.

On the other hand, body weight continued to decrease throughout the 6 months. This may reflect deficits in digestion which is destined to occur after total gastrectomy. It has been well documented that the main reason for body weight loss following gastrectomy is loss of adipose tissue [2, 3]. In contrast, LBM decreased by 3.4% at POM1 but recovered to the preoperative value at POM3. Since ghrelin is known to function as a growth hormone secretagogue [7, 8], we hypothesize the ability to preserve ghrelin levels following APG may have contributed to the maintenance of muscle mass despite overall weight loss. Although APG did not prevent postoperative weight loss, it may have a protective effect on muscle mass loss. Aoyama et al. [4–6] have reported that LBM loss was strongly associated with poor S-1 compliance. Thus, we expect that the preventive effect of APG on LBM loss may be important in terms of oncological prognosis.

Despite the aim of APG, a significant decrease in serum ghrelin level was observed at all postoperative time points compared to preoperative values. Nevertheless, 53% of the preoperative ghrelin levels were preserved at POM1, 53.5% at POM3, and 60.4% at POM6. This could be quite beneficial given that, according to a previous report, only 13% of serum ghrelin levels were preserved on day 7 after total gastrectomy, with no subsequent improvements [23, 24]. Further follow-up is necessary to clarify changes in serum ghrelin levels over time and to better understand the implications of preserving the ghrelin secretory portion of the stomach.

## Study limitations

The limitations of this study include the single-center, single-arm observational design of the study and the small number of patients analyzed. The advantage of preserving the ghrelin-secreting region has not been directly compared to that of total gastrectomy, and to what extent the ghrelin secreted from the residual stomach actually contributed to the preservation of appetite is uncertain. Moreover, even if the ghrelin-secreting region of the stomach is preserved, the absence of passage of food into the residual stomach as expected after APG may humper sufficient secretion of ghrelin. From that viewpoint, the relationship between food passage and ghrelin secretion needs to be investigated.

After exploring the feasibility of APG by minimally invasive approach in the latter part of the study, we therefore plan to proceed to the next step of confirming favorable nutritional consequences and assessing long-term oncological outcomes in a well-designed multi-institutional trial.

## Conclusions

APG can be safely performed, and the residual stomach functioned as an endocrine organ, resulting in higher serum levels of ghrelin, which in turn may have served to maintain appetite and preserve lean body mass despite a significant loss in the body weight.
